# “The Little Money I Get Is Used to Buy Drugs”: A Qualitative Exploration of the Economic Cost of Intimate Partner Violence for Female Survivors in Ghana

**DOI:** 10.1177/10778012231182408

**Published:** 2023-06-26

**Authors:** Gervin Ane Apatinga, Eric Y. Tenkorang

**Affiliations:** 1 7235 University of Saskatchewan, Saskatoon, SK, Canada; 2 Memorial University of Newfoundland and Labrador, St John's, NL, Canada

**Keywords:** Africa, economic cost, Ghana, women, violence

## Abstract

Empirical research confirms the economic costs of intimate partner violence (IPV) for women. Yet, scholarship on this topic is lacking in Ghana, where IPV against women is commonplace. We used in-depth interviews with 15 female survivors of IPV in the Eastern Region to examine the economic costs of IPV for women. Findings showed that the economic costs were both direct and indirect. Direct costs included out-of-pocket payments for medical and nonmedical services, while indirect costs included diminished work abilities, increased absenteeism from work, and lowered work productivity. Ghanaian policymakers must enforce and strengthen policies to prevent violence against women.

## Introduction

Intimate partner violence (IPV) against women is a substantial public health problem. Survivors of abuse report more physical injuries, psychological trauma, and emotional problems than their nonabused counterparts (Adu-Gyamfi, 2014; Apatinga et al., 2020; Chireshe, 2015; Sedziafa et al., 2016). They are also more likely to abuse drugs and alcohol and attempt suicide (Bonomi et al., 2009; Devries et al., 2011). Additional implications are economic, ranging from loss of days of work to lowered work productivity, diminished work abilities, and depletion of income (Duvvury et al., 2013; Merino et al., 2019; Vara-Horna, 2014). In a Ghanaian study, about 6 percent of abused women were found to have missed 11 days of paid work (Merino et al., 2019). Others have found female survivors struggle to concentrate at work, and this affects their productivity (Ridley et al., 2005). The economic impact of domestic abuse extends beyond the workplace. In some studies, abused women were found to use health care and social services more often and incurred out-of-pocket costs for medical and nonmedical services (Baum et al., 2009; Bonomi et al., 2009; Miller et al., 2007; Rivara et al., 2007). Bonomi et al. (2009) found that the health care costs of abused women were 42 percent higher than those of their nonabused counterparts.

As these findings demonstrate, exposure to male violence has economic implications for women. These, in turn, may lead to other adverse outcomes. For instance, they may create and reinforce gender inequalities, push women further below the poverty line, and prevent them from achieving economic independence and financial security. In this context, male partners are likely to capitalize on women's vulnerabilities (e.g., poverty) to manipulate and control them, consequently leading to violence.

Research on the economic costs of IPV for women is sparse in Ghana and sub-Saharan Africa. Studies have focused on the prevalence, drivers, and health implications for female survivors (Apatinga & Tenkorang, 2020; Apatinga et al., 2020; Tenkorang et al., 2013), with less attention to the economic impact on women's lives. Furthermore, research on this topic is largely quantitative, driven by the idea of quantifying economic costs (Asante et al., 2019) with little to no qualitative component to provide an in-depth understanding of the subjective experiences and perspectives of the women involved. Contributing to the sparse literature, we used qualitative data obtained from 15 female survivors of male partner violence in the Lower Manya Krobo District (LMKD) in the Eastern Region of Ghana to document the economic cost of IPV for women.

## Conceptual Framework and Empirical Literature

**Figure 1.**
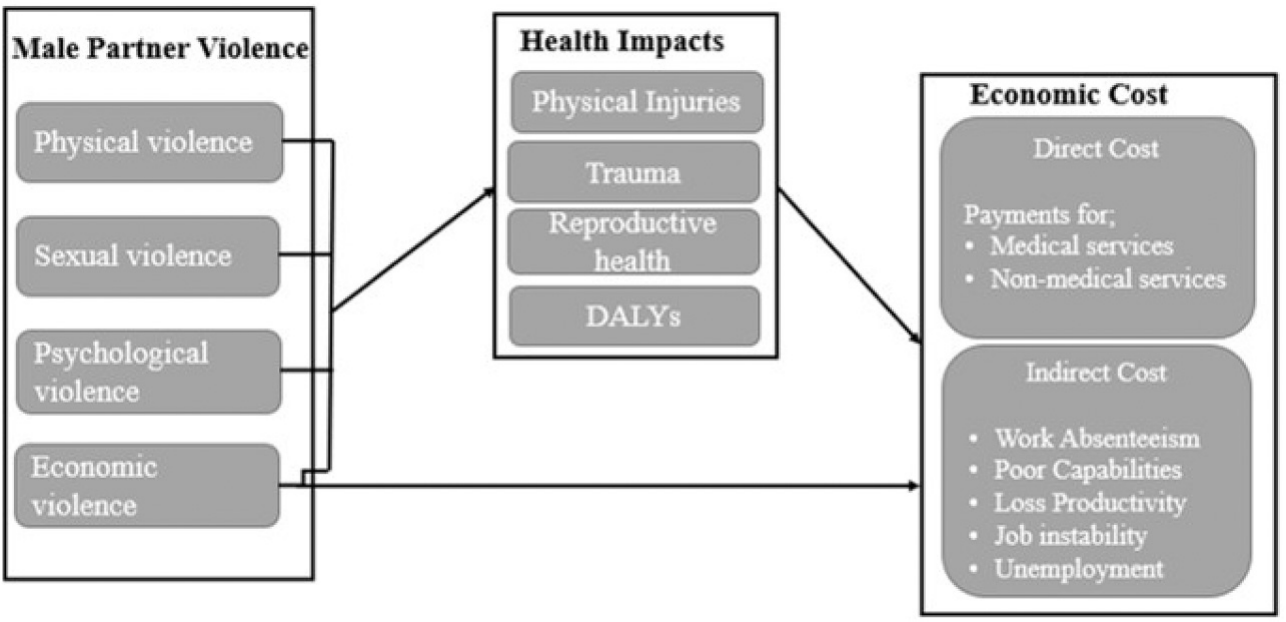
A conceptual framework outlining economic costs of IPV for women.

The study's conceptual framework (Figure 1) argues that IPV has significant economic consequences for women, broadly categorized as direct and indirect costs.

The direct costs refer to the real money women spend on medical and nonmedical services. The various types of male violence expose women to varied health problems. For instance, physical violence is associated with backaches, body aches, broken bones or dislocations, bruises, cuts, and pregnancy complications. Sexual violence and rape increase the risk for human immunodeficiency virus (HIV) and other sexually transmitted infections. Psychological violence exposes women to depression, posttraumatic stress disorders, insomnia, and nightmares. Economic violence threatens financial security and independence, leading to health problems such as anxiety or grief and insomnia.

In this context, women who experience at least one form of intimate partner abuse are likely to use health care services and social services more often, and this may incur direct out-of-pocket costs. In one study, the average medical care cost for female survivors of physical violence was $2,665 per incident (National Center for Injury Prevention and Control [NCIPC], 2003). In another study, sexually abused women were found to pay 29.2 percent of medical expenses and 33.6 percent of mental health costs out of pocket (Bonomi et al., 2009). Nonmedical services such as attorney fees and transportation to access medical and social services can be costly as well (Baum et al., 2009; Miller et al., 2007; WHO, 2008). Baum et al. (2009) found 12.9 percent of victims paid more than $1,000 for nonmedical services.

There are also indirect costs. These include but are not limited to diminished labor productivity and work absenteeism. IPV may affect women's ability to engage in income-generating activities and deprive them of economic resources. It can also have a debilitating impact on their health, thus determining how much time and energy they can spend on income-generating activities. They are likely to be more productive in the labor market when they have good health, but the effects of abuse can lead to loss of days of work and even to unemployment. If they must treat health complications such as physical injuries and trauma at health centers or seek help from the police and attorneys for assaults, their income-generating activities will suffer (Baum et al., 2009; Bonomi et al., 2009). They may lack concentration and perform poorly at work and miss work deadlines due to depression, fear, and trauma.

Suggestions that IPV affects women's labor force participation and productivity are backed up by the literature (Delisi et al., 2010; Johnson & Ferraro, 2000; Reeves & O’Leary-Kelly, 2007). In one study, Ridley et al. (2005) found injuries resulting from violence made almost 96 percent of IPV survivors less productive. The NCIPC (2003) reported IPV victims lose about 8 million days of paid work and 5.6 million days of household productivity. Female survivors who miss days of work have higher odds of losing their jobs or being forced to quit (Duvvury et al., 2012). Other studies have found economic abuse may deepen poverty for women and threaten their financial security and self-sufficiency (Adams et al., 2012; Schrag & Ravi, 2020; Sedziafa et al., 2017; Stylianou, 2018). Kutin et al. (2017) reported a positive correlation between financial stress and economic violence, a finding observed in other studies as well (Antai et al., 2014; Asencios-Gonzales et al., 2018).

The conceptual framework in Figure 1 shows exposure to male partner violence has complex and traumatic economic consequences for female survivors. It details the ripple effects of IPV on women and emphasizes the need to address this social problem through appropriate legislation and policies.

## Methods

### Study Setting

This study took place in the LMKD in the Eastern Region of Ghana, one of the regions with the highest prevalence of IPV in the country. In 2013, about 1,929 cases of IPV were recorded compared to 1,502 cases in 2012, a dramatic increase (Ghana News Agency, 2014). The LMKD has a total land area of 304.4 sq. km. (Institute of Development Studies, Ghana Statistical Services and Associates, 2016). The district is approximately 80 percent urban and was recorded as having a population of about 89,246 in the 2010 population and housing census (IDS, GSS, & Associates, 2016). Females outnumber males (53.5 percent and 46.5 percent, respectively). The majority of the people are Krobos, with a few other tribes, including Ewes, Akans, Hausas, and Guans (Ghana Statistical Service, 2014). The people are predominantly Christians (92.8 percent), with a few identifying as Muslims and traditional believers (Ghana Statistical Service, 2014). The Krobo people practice patrilineal inheritance, and women cannot own property (Kissi-Abrokwah et al., 2015).

The people are predominantly farmers, and the most common farming activities are roots and tuber crops and vegetable cultivation. Mangoes are also grown for local consumption and export purposes. Trading activities are common, with farm and nonfarm products traded as a source of livelihood. Fishing is another common activity. The area is endowed with natural resources, such as limestone, and has several tourist sites, including beautiful landscapes, the Kpong airfields, and beach resorts (IDS, GSS, & Associates, 2016). Despite these resources, poverty is widespread, especially among women (Otoo et al., 2009). LMKD experiences high rates of unemployment and HIV/acquired immunodeficiency syndrome (Ghana Statistical Service, 2013).

Women constitute more than half of the population, suggesting they contribute significantly to the socioeconomic activities of the area. They represent nearly half of the labor force and are found in almost every sector of the local economy, including agriculture, industry, and services. Their contribution to the formal and informal sectors strengthens the local economy. Yet, studies show an increase in the prevalence and incidence of domestic and marital violence in the area (Apatinga et al., 2020; Sedziafa et al., 2016; Sedziafia et al., 2017). As discussed previously, IPV has adverse health, social, and economic outcomes for women, limiting their full and equal participation in the development of the local and national economies.

In their study in the LMKD, Apatinga et al. (2020) found married women who suffered sexual violence experienced severe health complications, a finding confirmed by other studies in the area (Sedziafa et al., 2016; Sedziafa et al., 2018). Rates of IPV against women are unacceptably high in the Eastern Region of Ghana, where the LMKD is located. It is on this basis that we chose the LMKD to document the economic costs of IPV against women. It provided access to participants with the requisite knowledge and information on male partner violence to understand the economic costs for women.

### Study Participants

The sample comprised women with experiences of IPV in the LMKD. Participants were recruited after consulting the chief and his elders. Entering the community and building rapport were made much easier as the second author has long-established ties with the community. After we informed the gatekeepers about the purpose of the study and gained approval, we identified a few survivors of male partner violence through local opinion leaders, and we obtained their consent to participate in the study. The snowballing method was used to identify other participants through suggestions from the initial contacts. Minimum eligibility included women who were at least 18 years and had experienced domestic abuse and were legitimately married or cohabiting. Fifteen women survivors of IPV participated in the study, as Latham (2014) suggests a sample size between 15 and 20 homogenous participants is enough for many qualitative studies. Moreover, the point of saturation was reached, as participants started replicating similar stories in subsequent discussions.

### Data Collection and Instruments

Data were obtained in face-to-face in-depth interviews, as this method of data collection provides a more relaxed atmosphere for both interviewers and participants to discuss a topic of interest in detail (Boyce & Neale, 2006). In this way, participants were given a platform to share their experiences and perspectives on domestic violence. According to Clark, “the power dynamics of the interview situation is critical… The generation of an environment in which respondents feel relaxed and able to speak at length is therefore of fundamental importance to the qualitative interview” (Clark, 2017, p. 84). Participants suggested their preferred time and locations for the individual interviews.

Trained at the University of Ghana on how to conduct interviews, female research assistants began the interviews by explaining a detailed informed consent sheet that covered the aim of the study, issues of anonymity and confidentiality, and the right to discontinue the study without consequences, and then obtained participants’ consent. The interviews were held informally to create a more conducive atmosphere for both participants and interviewers to engage in frank and detailed discussions without intimidation. Participants were asked similar questions, and interviewers intermittently probed, when necessary, to clarify issues. A flexible script was used to moderate the interviews, where interviewers posed questions to participants to get them to voluntarily share their experiences and thoughts on domestic violence. This script was prepared in English but delivered in Krobo (widely spoken local dialect) for participants who did not understand and speak English well enough to respond to questions adequately. The script covered such topics as sociodemographic characteristics, prevalence and forms of IPV, perceived causes and consequences, responses to violence, and survivors’ support-seeking behaviors.

Each interview lasted approximately an hour, as this time frame allowed meaningful and exhaustive discussions. With permission from participants, in-depth interviews were audio-recorded to reduce distortions and misrepresentation of responses. For easy transcription and analysis of the data, the recorded interviews were listened to multiple times to obtain a complete, unbiased, and objective analysis and representation of results. The interviews in English and Krobo (interviews in the local dialect were back translated into English) were then transcribed verbatim into soft copy. IDs were used to mask participants’ personal information.

### Ethical Considerations

This study was part of a larger project approved by the Ethics Committees at Memorial University (Interdisciplinary Committee on Ethics in Human Research) and University of Ghana (University of Ghana Ethics Committee for Humanities). Trained female research assistants obtained informed consent from all participants in written and oral forms. Participants were also informed about the right to withdraw from the study at any time without consequences. The principles of the safety of information and the protection of the identity of participants were rigorously adhered to in the data collection and transcription. All interview transcripts and other data were alphanumerically coded when the scripts were being analyzed. Standby counseling services were available to help stabilize victims of violence who were distressed by recounting their experiences of violence. The World Health Organization's (2001) ethical and safety recommendations on IPV guided this study.

### Data Analysis

We used content analysis, as this technique is crucial for describing in detail people's perspectives on and experiences of a topic (White & Marsh, 2006). We began the analysis by devoting ample time to reading the transcribed text, as researchers are urged to invest time and engage with interview transcripts (Kvale & Brinkmann, 2009). Investing time allowed us to familiarize ourselves with the data, gain a deeper understanding of women's responses, recap the social and emotional aspects of the discussions, and ascertain the limitations of the data.

We then used QDA Miner, a computer analytical tool, to code and analyze the transcribed text. The transcribed text was coded inductively to find common ideas and patterns. The generated codes helped us to identify meaningful categories and subcategories. These categories were then synthesized into broader themes based on the objective of the study and used to present women's experiences of and perspectives on the economic costs of IPV. Inductive data coding and assigning of text to codes were undertaken by the first author and supervised by the second author to check the findings’ consistency, accuracy, and reliability.

## Findings

### Demographics

Participants’ ages varied widely, from 25 to 65 years. Nearly all were married or living with their male partners. Almost all were Krobos, with one woman identifying as Ewe. Participants’ educational attainment varied significantly: four completed some secondary education level, two attained primary education, one completed tertiary education, and a significant number had no form of education. They were all Christians. The number of children born to each participant ranged from 1 to 10. Participants engaged in different economic and livelihood activities. Most were petty traders, and a few were farmers. One participant identified as a professional teacher.

### Economic Costs of IPV for Women

Participants reported various economic difficulties after experiencing male partner violence. These are discussed under the following broad themes: direct and indirect economic costs.

#### Direct Economic Costs

Our participants told us they spent a significant part of their earnings paying for not only medical but also nonmedical services.

##### Medical Expenditures

Participants said they experienced physical injuries and psychosocial problems after suffering from male partner violence. Some sustained injuries after forced sex, such as body aches and headaches. They also reported posttraumatic stress disorders, depression, distress, and nervousness. Some who were pregnant experienced miscarriages. Their precarious circumstances meant they often spent their daily earnings and profits on medical expenses. Many participants said health care utilization and buying antibiotics were common in their lives, as they frequently experienced all forms of IPV. They explained this was expensive, especially for poor women.

Two of our participants said the following about the medical expenses they incurred after experiencing IPV:

My eyes were swollen for days, and I had severe pains around my neck and had to go to the hospital. I had to do that with my own money since my husband totally avoided me. I was not able to go to the market for days, so I lost so much money. The few days I went, the little profit I made was used in buying drugs. (Alimah, 37 years)

Some of the beatings affected me so much that I had to buy drugs to treat myself. Sometimes I feel very sick the morning after the night he beats me. There was an instance where he got very angry and took the iron that was lying on the ironing bed closer to the bed and threw the iron at me. It hit my head I felt a sharp pain in my head. It became so severe that I had to go to the hospital. I went and paid money, and they treated, but once in a while I feel the pain. (Fosia, 28 years)

##### Nonmedical Expenditures

It also became clear from participants’ accounts that they incurred costs traveling to and from hospitals and clinics. Some mentioned the cost of traveling to police stations and filing complaints with legal authorities. Some told us the high cost of seeking help from formal support units often discourages survivors from reporting their abusive husbands. The following extracts describe these concerns:

When you go to the police to report, you are going to buy paper, and you will buy pen and maybe you are not having money to even buy the pen. But immediately you inform the elders, they sit down to settle the dispute and advise each of the parties without you paying anything. They will not be postponing and tossing you around; they will settle it for you. But the police will postpone, you will buy a pen, you will pay for transportation to the police station, and all this is a waste of time and drains resources. (Fuseina, 48 years)

I once reported my husband to the police. I went to the Odumase police station to report my husband's actions towards me. I was asked by the policemen to go to Akuse where domestic violence issues were addressed, but I was not working; therefore, transportation even to Akuse was a problem. (Latifa, 26 years)

#### Indirect Economic Costs

Participants said IPV made it difficult for them to perform their economic activities. Physical and psychological trauma and partner interference hampered their work. Physical, psychological, and financial abuse also caused job instability. Commonly cited difficulties were lost opportunities, lost income, lost time to work, and lowered productivity. Productive working hours were lost to treating health complications resulting from IPV. For instance, participants who constructed their livelihoods on farming said they hardly ever visited their farms when they were healing from their health complications. Other participants who based their livelihoods on nonfarming activities infrequently attended to their businesses following episodes of IPV. These circumstances depleted their incomes and destabilized their livelihood activities.

The following quotations clarify how IPV made it difficult for participants to perform their farm and nonfarm activities:

When he slapped me, my face became swollen. The second slap and the beatings were very serious. There were marks all over my body. I could not go to work for about two weeks because of the marks on my face and I also did not want people to know so I hid in my room to prevent people from asking questions. I lost a lot of money and some of my customers because of how my husband has been beating me. (Ayishetu, 43 years)

My face was swollen for days. I could not go out for days; people were asking me what had happened. Due to the pain, I could not go to work for two weeks. My husband realized it was serious, so he gave me money to go to the hospital, but I did not go. I went to buy drugs from the pharmacy, and I became better. So the money I could have made within these two weeks I lost it. (Memunatu, 56 years)

My husband started maltreating me, and others blamed me for what had happened to me. I fell sick and was admitted at the Atua Government hospital for two weeks. After I was discharged, I was not fit so for about two months I could not work. It affected my finances very much. (Asana, 55 years)

Some participants said financial control and exploitation restricted them from accessing opportunities and engaging in businesses to better their lives. The following are some of their personal stories:

My husband does not want me to work; he believes when I begin to work, I will be able to buy more attractive clothes. He thinks this will make me attracted to other men. He insisted I wear very long dresses everywhere and prevented me from wearing other clothes. He also does not like when friends visit me or when I go out with friends. He always wants me to be indoors. He does not support me financially; he has refused to give me money to start a business of my own and does not often give me housekeeping money. He also insults me anyhow he likes. (Rabiatu, 25 years)

He stopped giving me housekeeping money. He moved to his mother's room and left us alone. He later told me to move to my father's house for six months, so we come back but I refused. He tried convincing me to leave but I refused. He insulted my family on top of his voice in the house and disgraced me in public. He restricted me on how I use my money and made me use all my money to buy cement and blocks which was used to renovate their house. He also sometimes steals my money when I bring the money home. (Rahama, 28 years)

My husband goes to borrow money from people and gives them my number that they can call me for the money. Some call to insult me because he does not pay them back their money. It got to a point I had no money in my account because he drained me seriously. People around and relatives knew I was working so they were also demanding from me, but I had nothing to give them. I couldn’t get some little monies to take care of my needs. (Alimah, 37 years)

The depletion of income and job instability arising from consistently missing work schedules, reduced productivity, and marginal work commitment compelled some women to resort to borrowing money from friends, families, and corporate entities to boost their businesses. But borrowing money had repercussions:

When my husband beat me, it affected all aspects of my life. My little business suffered so much because I was spending more than I was earning. The things I was selling got finished and I had no money to buy them. All my customers stopped buying from me because when they come, they do not get what they want to buy. I borrowed money from most friends and family members, and I could not pay some of them. So they came to the house and embarrassed me. I could not relate with them as I used to because they no longer had any respect for me. (Abiba, 50 years)

My husband's behavior worries me so much because I work more than necessary just to take care of our children. I used any money I get to take care of our children. They need money for books, fees, and others and my husband has decided not to take care of them. I am suffering very much in the hands of this man because sometimes I have to borrow money from friends just to put food on the table or make sure my children are in school. He misuses money when he gets some and when there is no money at home, he does not make any attempt to provide food for the family. This whole month he has been complaining that he does not have money on him, so I even have to pay his transportation every day to work. (Fosia, 28 years)

It is clear from participants’ accounts that their paid work was affected by violence: they lost both income and savings.

## Discussion

Although IPV against women is well-discussed and is known to have deleterious consequences for female survivors, some critical aspects are underresearched and less documented in Ghana. In particular, little attention has been given to the economic implications of IPV for women despite growing evidence that male violence may have devastating economic consequences. To augment the scant literature, we analyzed qualitative data collected from 15 female survivors of male partner violence in the Eastern Region of Ghana to explore the economic costs. The content analysis showed the costs women incurred were both direct and indirect. Direct costs included personal payments for medical services and nonmedical assistance. Indirect costs were job instability, reduced productivity, marginal commitment to work, and missed work schedules. These costs significantly undermined women's economic activities and depleted their finances.

### Direct Economic Costs

Consistent with previous research (Arias & Corso, 2005; Bonomi et al., 2009; Duvvury et al., 2004; Rivara et al., 2007), we found women in the LMKD experienced direct economic costs after suffering from male partner violence. IPV exposes women to multiple intersecting health risks and vulnerabilities. They are more likely to visit and use health care services more frequently, and they often pay their expenses. Most of our participants said they spent money for medical services such as hospital bills and medications and social services such as transportation following IPV, for example, “I felt sick for days and had to go to the hospital to seek medical attention; I used my own money to buy drugs and even pay my hospital bills” and “The little money I get is used in buying drugs.”

In cultures that accept or even promote violence, women may have to spend their own money to get help, and they may have to do so more often: in a snowball effect, the health-related impacts of IPV increase health care utilization, leading to higher out-of-pocket costs. Over time, female survivors are likely to lose vast sums seeking medical treatment. This will be especially hard on poor and marginalized women. It may profoundly affect household consumption, preventing women and their families from accessing essential goods and services or meeting their basic needs. Coupled with less money coming into the household, being forced to pay to recover from the health impacts of IPV may drain women's financial resources and lead to financial insecurity, thus worsening their living conditions.

The findings demonstrate interlinkages between IPV, poverty, and gender inequalities. Addressing domestic and marital violence in this context is essential to allow women to build their capabilities and become empowered.

### Indirect Economic Costs

Like previous research (Duvvury et al., 2013; McLean & Bocinski, 2017; Modi et al., 2014), we found male partner violence affected women's ability to perform their income-generating activities and deprived them of economic resources. Our participants were unproductive in the labor market because of health complications. The effects of physical, sexual, and psychological abuse caused them to lose days of work. They spent time treating health problems and seeking help from formal support units such as the police at the cost of their income-generating activities. Financial exploitation and control and partner interference further restricted them from accessing resources and opportunities to engage in business. Participants also said they could not concentrate; they underperformed at work and missed work schedules because of depression and trauma resulting from IPV. Our findings were not surprising, as previous research has found strong associations between male partner violence and loss of days of paid work, lowered productivity, and unemployment (Baum et al., 2009; Bonomi et al., 2009; Delisi et al., 2010; Johnson & Ferraro, 2000; NCIPC, 2003; Reeves & O’Leary-Kelly, 2007; Ridley et al., 2005).

A critical look at our participants’ narratives showed the higher the occurrence and severity of male violence, the more likely women were to experience issues of absenteeism (missing days of work to address physical and mental health and legal issues), presenteeism (difficulty concentrating on tasks, working slowly and stopping work despite being physically present), and tardiness (taking time off work to address conflicts at home and regain calm before returning to work), findings also reported elsewhere (e.g., Alexander, 2011; Lohaus & Habermannb, 2019; Swanberg & Logan, 2005). When women's economic independence and financial security are undermined, they may resort to borrowing money, leading to debt accumulation and poor credit. Such precarious situations may be more common in environments where IPV is widespread—as in our study area and among our sample. More women in these environments may be forced into poverty. IPV has been found to disempower women, push them further below the poverty line, and increase their financial stress (Adams et al., 2012; Schrag & Ravi, 2020; Sedziafa et al., 2017; Stylianou, 2018). It may make women less autonomous and reduce their agency, compelling them to depend on their male partners for financial support and put up with repeat assaults.

These cooccurring experiences may push women to attempt suicide as a means of escape. The loss of autonomy, heightened levels of hopelessness, economic disempowerment, and the inability to escape rancorous relationships may compel women to commit suicide or attempt suicide. A growing literature demonstrates the relationship between IPV and female suicidal behavior (Crosby et al., 2011; Gold et al., 2012; Logan et al., 2011). Although none of our participants mentioned this possibility, it should be a concern. It underscores the need to create violence intervention programs.

## Implications of the Findings

To the best of our knowledge, this is one of only a few studies in Ghana to document the economic costs of IPV for women. Using qualitative data to examine economic costs offers a different perspective on this topic. While there are calls to end IPV because of human rights abuses and public health problems, our findings show the economic consequences for survivors, reinforcing the need to design policies and programs to decrease rates of violence against women. They also suggest the need for further research in different local contexts. Such studies will strengthen arguments to engage governments and stakeholders in intervention programs, as policymaking and programming are currently evidence-based.

## Limitations

Despite the policy relevance of the findings, some weaknesses should be acknowledged. Using only 15 qualitative in-depth interviews limits our ability to generalize the findings to the larger population. Moreover, considering the sensitivity of IPV issues in the Ghanaian context, we could have encountered social desirability bias. Participants may have overreported or underreported their experiences of IPV and its associated economic costs. Nevertheless, the narratives provide a detailed understanding of the economic costs of IPV and call for increased scholarly and policy attention.

## Conclusion

Despite the passage of laws such as the Domestic Violence Act in Ghana to protect against violence in domestic relationships, rates of IPV remain unacceptably high in the Ghanaian female population. Prior research underlines that females are overrepresented in domestic violence cases. Approximately one-third of Ghanaian women are likely to experience any form or a combination of several forms of IPV in their lifetime. Despite its prevalence, some critical aspects of male violence toward women have received less scholarly attention. In particular, little is known about the economic implications for women.

In the context of national and international efforts to reduce violence against women, promote gender equality, and empower women, this study explored the economic consequences of IPV for women in Ghana to support prevention and mitigation programs and policies. Findings showed that IPV was commonplace in our participants’ lives, and the economic costs they experienced were direct and indirect. Personal payments for medical and nonmedical services were the direct costs identified. The indirect costs were not actual expenditures but opportunity costs in reduced productivity. Participants reported a range of consequences, including depletion of personal incomes, poor development of capabilities, and economic stress.

This study broadens our knowledge of the devastating consequences of male partner violence, especially in an area where academic research is lacking. While the topic requires more scholarly attention, the results show that IPV has severe economic implications for women. There is a need to create and enforce a broader set of policies and programs to prevent violence against women. The etiology of male partner violence must be eliminated through effective legal instruments and policy frameworks. In collaboration with other stakeholders, the government of Ghana must assiduously enforce laws against domestic violence and respond to incidents of abuse. This would include revising and updating existing legal instruments and policy frameworks with new information on IPV.

Moreover, men and women must be educated through the media and in community educational programs sensitizing them on how IPV affects both individual women and the broader society. Regular media discussions would enlighten people about the damaging consequences of IPV; this could help reduce the level of violence against women. Efforts must be made to dismantle patriarchal structures of hegemonic masculinity and address attitudes and behaviors that lay the groundwork for marital conflicts. Further, addressing IPV against women must move beyond lip service to include practical approaches to dismantle structures that underlie and support gender inequalities and poverty—significant risk factors for IPV. In short, women's experience of male partner violence is complex and traumatic, making the development and enforcement of policies, programs, and interventions to address this social problem particularly compelling.
